# Dosimetry of a Thermoregulated TEM Cell for 5G 700 MHz and 3.5 GHz Band Frequencies for Bioelectromagnetic Investigations

**DOI:** 10.3390/s26082393

**Published:** 2026-04-14

**Authors:** Abdelkhalek Nasri, Lionel Michard, Lena Serradeill, Rosa Orlacchio, Yann Percherancier, Philippe Leveque, Claire Dalmay, Delia Arnaud-Cormos

**Affiliations:** 1 XLIM, UMR 7252, CNRS, University of Limoges, 87060 Limoges, France; abdelkhaleknasri2012@gmail.com (A.N.); lionel.michard@unilim.fr (L.M.); philippe.leveque@unilim.fr (P.L.); claire.dalmay@unilim.fr (C.D.); 2 IMS, UMR 5218, CNRS, University Bordeaux, INP, 33400 Talence, France; lena.serradeill@u-bordeaux.fr (L.S.); rosa.orlacchio@ephe.psl.eu (R.O.); yann.percherancier@ims-bordeaux.fr (Y.P.); 3 École Pratique des Hautes Études (EPHE), Paris Sciences et Lettres Research University, 75014 Paris, France; 4 Institut Universitaire de France (IUF), 75005 Paris, France

**Keywords:** transverse electromagnetic (TEM) cell, dosimetry, radiofrequencies, 5G frequencies, thermoregulation

## Abstract

**Highlights:**

**What are the main findings?**

- The design and dosimetry of wide-bandwidth thermoregulated transverse electromagnetic cells for bioelectromagnetic investigations.

**What are the implications of the main findings?**

- An RF exposure system for 5G-band signals—700 MHz and 3.5 GHz—applied to bioluminescence resonance energy transfer cellular studies.

**Abstract:**

This work presents the design and characterization of a thermoregulated, bandwidth-enhanced TEM cell system optimized for bioelectromagnetic experiments on biological cells, with a focus on bioluminescence resonance energy transfer investigations at 700 MHz and 3.5 GHz. Bandwidth improvement, achieved through geometric modifications and optimized connector transitions, resulted in reduced return and insertion losses and improved field uniformity, particularly in the 2.5–6 GHz range. Numerical simulations showed homogeneous electric field and normalized specific absorption rate (SAR) distributions (~1 W/kg) at 700 MHz. At 3.5 GHz, the improved TEM cell provided the most uniform exposure of the biological sample with SAR values of 15 W/kg and 10.5 W/kg, for the bulk and surface (bottom layer), respectively. Experimental SAR measurements using a ~1 mm^3^ fluoro-optic probe agreed well with simulations. To counteract RF-induced heating, the system incorporated active thermoregulation at 37 °C. At 3.5 GHz and 20 W input power, a 1.5 °C rise over 120 s was effectively mitigated using water-circulation cooling. This work provides a controlled and reliable setup for future studies on the interaction of 5G-band electromagnetic fields with biological systems.

## 1. Introduction

Transverse electromagnetic (TEM) cells were first introduced by M. L. Crawford in 1974 [1]. Since then, TEM cells have been widely employed to perform electromagnetic compatibility (EMC) and electromagnetic interference (EMI) testing. Moreover, TEM cells also play an important role in the bioelectromagnetic field, as they provide a polyvalent and straightforward solution to expose in vivo samples to electromagnetic fields (EMFs). Several studies featuring TEM cells dedicated to the assessment of the effects of EMFs on biological cells have been reported in the literature [2,3,4,5,6,7,8]. Notably, TEM cells can be integrated into experimental setups dedicated to the study of the potential activation of several ion channels through bioluminescence resonance energy transfer (BRET) [9].

One of the main drawbacks of TEM cells lies in their bandwidth limitation, in particular for high frequencies associated with new mobile technologies, such as 5G sub-6 GHz, identified as FR1. Indeed, as the frequency increases, higher-order propagation modes and resonances arise. Additionally, localized impedance mismatches, e.g., near the connector transitions, become more significant. A common strategy to mitigate these limitations consists of reducing the dimensions of the TEM cells, as this naturally shifts higher-order modes and resonance cutoffs towards higher frequencies [1,10,11]. However, some applications, such as BRET investigations, require an incompressible test volume, thus limiting size modifications. Several approaches to enhancing TEM cell bandwidth have been presented. For example, studies conducted by D. A. Hills [12] and R. Lorch [13] allowed for the enhancement of the bandwidth of TEM cells by focusing on mode suppression from 25 to 45 MHz and from 450 to 670 MHz, respectively. Sh. Deng et al. [14,15] investigated numerous designs to suppress higher-order modes without changing the test volume. T. Zhou et al. [16] presented a new TEM cell structure for wider bandwidth, and G. Giannetti [17] employed the septum tapering technique to minimize reflections near the connector transitions. Although the proposed designs employ an absorber or comparable methods to broaden the applicable frequency range, they consequently reduce the usable test volume or require the use of complex and sizable materials. Other studies focusing on impedance matching in a limited standard bandwidth are reported in [18,19]. Currently, suppression of higher-order modes in TEM cells remains challenging but persists as a relevant topic due to the prohibitive cost and complexity of other EMC test environments. This has motivated the investigation of geometry-based optimization strategies proposed in this work, which aim to enhance the bandwidth of TEM cells while preserving both the usable test volume and the electric field uniformity.

BRET is a spectroscopic biophysical technique that enables real-time measurements of protein conformational changes and protein–protein interactions in living cells. BRET was selected as the functional readout because the reference technique, i.e., patch-clamp electrophysiology, is inherently difficult to implement under radiofrequency (RF) exposure. The patch-clamp technique relies on highly sensitive low-noise measurements. RF electric fields inevitably introduce noise and artifacts into the electrochemical recording chain, degrading the signal-to-noise ratio. In this context, BRET offers a robust alternative for monitoring real-time ion channel activity during RF exposure, enabling discrimination between thermal RF effects and the potential non-thermal modulation of thermo-transient receptor potential (TRP) channels. Because RF exposure can induce dielectric heating in aqueous media, temperature must be tightly controlled when using BRET to probe thermo-TRP activity. Indeed, even small RF-driven temperature drifts can directly gate thermo-TRPs and thus mimic non-thermal effects in the BRET signal.

In this work, our objective is to design an exposure system capable of delivering elevated exposure levels relative to international guidelines (ICNIRP [20] and IEEE [21]), with strict control of temperature rise to prevent confounding thermal effects. A TEM cell-based thermoregulated experimental setup operated at 700 MHz and 3.5 GHz dedicated to BRET investigations is presented. The TEM cell bandwidth enhancement was achieved by improving impedance matching near the connector transitions and by reducing the width of one of the outer conductors. Details on the TEM cell structure and its electromagnetic characteristics are provided. Additionally, a dosimetry investigation, presented in Section 3, was conducted to assess the specific absorption rate (SAR) within the biological samples placed in the TEM cell test volume.

## 2. Materials and Methods

### 2.1. TEM Cell

**Figure 1:**
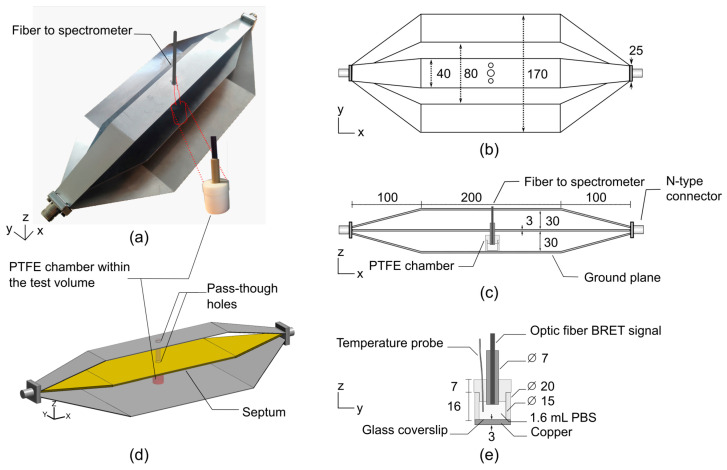
Schematic representation of the improved TEM cell-based exposure system dedicated to BRET investigations. Dimensions are given in mm. (**a**) Photograph of the manufactured stainless-steel TEM cell and PTFE chamber. Two 10 mm diameter pass-through holes were drilled into the septum and top of the TEM cell to allow for the insertion of an optical fiber for BRET signal reading. (**b**) Top and (**c**) side views of the TEM cell, including the PTFE chamber within the test volume. (**d**) 3D view of the simulated TEM cell and PTFE chamber. (**e**) Cross-sectional view of the PTFE chamber filled with 1.6 mL of phosphate-buffered saline (PBS) solution.

Similar to strip-line transmission lines, TEM cells are composed of three parts, i.e., a central connector (septum) and two outer conductors (ground planes). At each end, the structure is typically terminated by 50 Ω impedance-matched connectors. The proposed bandwidth-improved TEM cell is shown in Figure 1. A polytetrafluoroethylene (PTFE, Sigma-Aldrich, Saint Louis, MI, USA) chamber filled with 1.6 mL of phosphate-buffered saline (PBS, Sigma-Aldrich, Saint Louis, MI, USA) solution was positioned on top of the lower outer conductor of the TEM cell to perform BRET investigations. Two 10 mm diameter pass-through holes were drilled into the septum and top of the TEM cell to allow for the insertion of an optical fiber for BRET signal measurements.

With this novel TEM cell design, we propose an innovative approach to shift the unwanted higher-order propagation modes and resonances towards higher frequencies. In the study conducted by P. F. Wilson and M. T. Ma [10], a rigorous semi-analytical model is presented to assess the higher-order mode cutoff and resonant frequencies in TEM cells. According to the introduced formulas, it appears that there are three relevant dimensions in the design of TEM cells that can strongly affect the cutoff frequencies. The three key parameters describing the performance of TEM cells in terms of bandwidth are the distance between the outer conductors and the septum, the width of the septum, and the width of the outer conductors. Without altering the usable test volume, reducing the width of the upper outer conductor was found to significantly improve the bandwidth. Thus, comprehensive numerical simulations were employed to assess the optimal width of the upper outer conductor. As shown in Figure 1a,b, the optimal width was found to be 40 mm.

### 2.2. BRET Investigations Setup

**Figure 2:**
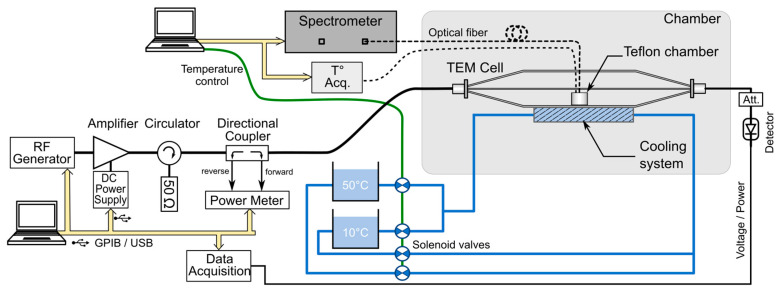
Schematic overview of the experimental BRET investigation setup based on the improved TEM cell. A cooling system coupled with a temperature measurement system allows for precise closed-loop thermoregulation within the PTFE chamber, where the cells are exposed.

The experimental setup dedicated to BRET investigations based on the bandwidth-improved TEM cell is shown in Figure 2, and a detailed list of the components is given in Table 1. A PTFE chamber was positioned on top of the lower outer conductor of the TEM cell. To enable BRET signal measurements, two circular apertures of 10 mm diameter were drilled in both the septum and the upper outer conductor of the TEM cell, allowing for the insertion of the optical fiber probe without significantly disturbing the electromagnetic field distribution.

The RF signals, provided by a vector signal generator, were amplified by a 100 W power amplifier and transmitted to the TEM cell through a 30 dB directional coupler. Closed-loop temperature control of the cell culture medium was achieved by employing a water-cooling system positioned beneath the lower outer TEM cell conductor.

BRET signals were recorded in real time using an optical-fiber-based detection system optimized for live-cell measurements under simultaneous temperature control (Figure 2). Full emission spectra were acquired with a fiber-coupled spectrometer (Table 1), as previously described in [6]. All acquisition parameters, including temperature feedback, RF-EMF exposure timing, and BRET acquisition, were synchronized through the same custom LabVIEW (version 2019, National Instruments, Austin, TX, USA) interface.

Thermoregulation was achieved using a water-circulation system thermally coupled to the metallic base of the exposure device (Figure 2). During sham conditions, a thermostated bath set at 37 °C was circulated through the reservoir located beneath the cell samples, ensuring efficient heat exchange with the culture chamber through the copper bottom. Upon RF exposure, a secondary bath maintained at a lower temperature was used as an auxiliary cooling source and could be activated through solenoid valves.

The temperature difference between the 37 °C bath and the cold bath was adjusted to counteract RF-induced dielectric heating and maintain the culture temperature close to the target value throughout the exposure period.

Culture temperature was continuously monitored using a fluoro-optic thermometer (Table 1), with the probe placed in direct contact with the cell monolayer to provide an accurate measurement of the sample temperature throughout the experiment.

### 2.3. Dosimetric Analysis

To quantify exposure conditions, numerical simulations and experimental dosimetry were performed, enabling the accurate determination of the specific absorption rate (SAR). Numerical dosimetry was carried out using a custom finite-difference time-domain (FDTD) code that solves Maxwell’s equations in the time domain within a spatially meshed structure composed of elementary cells. The simulated geometry is shown in Figure 1d.

The PTFE chamber material was modeled with a relative dielectric permittivity of 2.1. The biological medium, i.e., 1.6 mL of PBS solution, was modeled using the electrical properties listed in Table 2, which were obtained from experimental measurements and applied in numerical simulations at 700 MHz and 3.5 GHz (exposure frequencies).

Experimental dosimetry was carried out based on temperature measurements using the fluoro-optic temperature probe. Within this context, SAR is given by

$$SAR=c{\left.\frac{dT}{dt}\right|}_{t=0}$$

where T (K) is the measured temperature, c (4186 J/(kg K)) is the specific heat capacity of the cell culture medium, and t (s) is time. This methodology allowed for the quantitative assessment of RF energy dissipation in the samples, ensuring reproducibility across experiments and providing a benchmark for comparison with numerical dosimetry results.

Both previously designed [9] and bandwidth-improved TEM cells were characterized in the frequency domain by measuring the scattering parameters, S_ij_, i.e., reflection and transmission coefficients, with a vector network analyzer (HP 8722D, Agilent, Santa Clara, CA, USA), presenting a 50 Ω test port impedance.

### 2.4. Parameters of the Numerical Modeling

Given the large dimensions of the TEM cell relative to the small size of the PTFE chamber, a nonuniform spatial discretization was employed, incorporating a 167 μm fine-mesh grid. FDTD simulations must employ a spatial resolution at least ten times finer than the minimum wavelength to ensure numerical stability and accuracy [22,23,24]. The discretization settings were selected to ensure a sufficiently fine spatial resolution within the biological sample, corresponding to a mesh size of approximately λ/60 in all three spatial directions. The maximum cell size was 0.5 mm along the y- and z-axes and 2 mm along the x-axis. Overall, the FDTD computational domain consisted of a spatial grid of 829 × 205 × 297 elementary mesh cells. A spatial symmetry along the y-axis was used to reduce the computational volume. The upper limit of the increment time step (Δ*t*) is related to the size of the spatial mesh (Δ*x*, Δ*y*, Δ*z*), as imposed by the Courant stability criterion:

$$\Delta t\leq \frac{1}{c\sqrt{\left(\frac{1}{{\Delta x}^{2}}+\frac{1}{{\Delta y}^{2}}+\frac{1}{{\Delta z}^{2}}\right)}}$$

where *c* is the celerity of electromagnetic waves in free space, and Δ*x*, Δ*y* and Δ*z* correspond to the smallest mesh grid. The number of iterations was 50,000, representing a total simulation of 14.5 ns, resulting in a time step of 0.29 ps.

To prevent electromagnetic wave reflection at the boundaries of the computational domain, perfectly matched layers (PMLs) were added to the domain [25]. The number of PML layers was set to 18. This robust FDTD code has been validated and routinely employed over the past two decades for the design and dosimetric assessment of numerous exposure systems.

## 3. Results

### 3.1. TEM Cell Frequency and Matching Bandwidth Characterization

In this section, we compare the improved TEM cell presented in Section 2.1 against a “standard” 170 mm wide TEM cell, i.e., without upper outer conductor width reduction. In the following, the two TEM cells are denoted as “standard” and “improved” (40 mm wide with respect to the width of the upper conductor).

**Figure 3:**
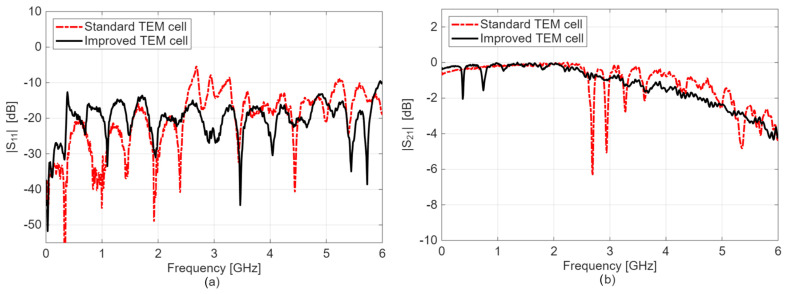
S-parameters results extracted from experimental measurements using a VNA for the standard and improved cells: (**a**) reflection coefficient, |S_11_| in dB; (**b**) transmission coefficient, |S_21_| in dB.

Figure 3 shows a comparison of the measured S-parameters. As observed, for the narrow TEM cell, the reflection coefficient magnitude, i.e., |S_11_| in dB, is inferior to −10 dB up to an approximately 6 GHz frequency range, indicating that less than 10% of the forward power is reflected at the TEM cell input. The criterion of ∣S_11_∣ < −10 dB was selected because it is widely used as a standard threshold in radiofrequency and microwave engineering, including in many bioelectromagnetic exposure systems. Except for two discrete frequencies in the 0–1 GHz bandwidth, the transmission coefficient, i.e., |S_21_| in dB of the improved cell, ranges between 0 and −1 dB up to 3.5 GHz, proving that more than 89% of the incident power is effectively transmitted through the structure. Conversely, the transmission coefficient, |S_21_| in dB of the standard TEM cell, exhibits resonant frequencies between 2.6 and 3.8 GHz, and the improved cell demonstrates a rather flat transmission curve. For example, for the standard TEM cell, the first significant impedance mismatch appears around 2.6 GHz, with a return loss of −5.7 dB (|S_11_| in dB) and an insertion loss of −6.3 dB (|S_21_| in dB). Conversely, at the same frequency, the improved TEM cell exhibits return and insertion losses of −16 dB and −0.8 dB, respectively. At 3.5 GHz, the return and insertion losses in the improved cell were approximately −25 dB and −1 dB, respectively.

In the standard TEM cell, resonant TE_11p_ modes with *p* = 2–5 were observed in the simulation at 2.70, 2.94, 3.28, and 3.61 GHz, respectively. In contrast, none of these resonances appeared in the improved TEM cell. This indicates that reducing the width of the upper outer conductor suppresses the propagation of the TE_11_ mode, thereby eliminating the associated resonances.

To summarize, the improved cell demonstrates superior performance in the 2.5–6 GHz band compared to the standard one, mainly due to the suppression of resonance frequencies. These findings confirm the applicability of the approach introduced in Section 2.1, consisting of reducing the width of the upper outer conductor of the TEM cell.

### 3.2. Electric Field Profile

**Figure 4:**
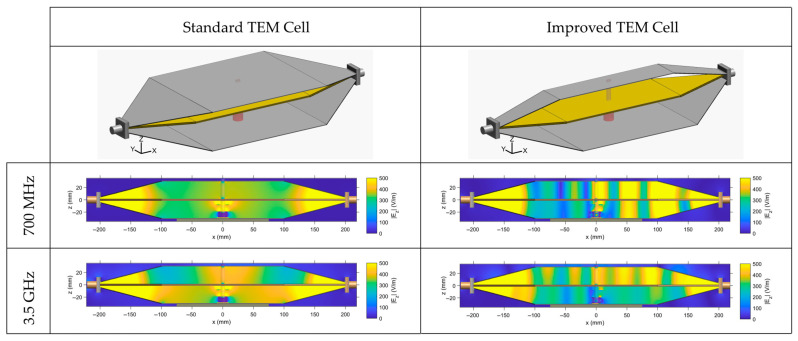
Electric field profile extracted from numerical simulations within the standard and improved TEM cells at 700 MHz and 3.5 GHz. Plotted distribution corresponds to the z component of the electric field in the xOz cut plane along the center of the structure.

From numerical simulations, we have extracted the electric field profile for the standard and improved cells at 700 MHz and 3.5 GHz (Figure 4). The distribution is extracted along the center of the structure for the main electric field component, E_z_.

As observed, at 700 MHz, the electric field is rather homogenous for the standard TEM cell with a symmetrical distribution along the TEM cell height, whereas the improved cell presents some electric field inhomogeneity. Indeed, the latter illustrates the presence of a slight standing wave within the TEM cell structure associated with the higher insertion losses measured at 700 MHz (approximately −1.5 dB).

At 3.5 GHz, overall, both TEM cells present electric field inhomogeneities. However, the improved cell is rather homogenous in the test volume between the septum and the bottom outer ground conductor. This volume corresponds to the area where biological samples are placed in the PTFE chamber for BRET investigations (Figure 1).

Table 3 indicates field variation expressed in ±dB relative to the mean and corresponding to the time-averaged field within the biologically relevant volume. It characterizes the electric field distribution, for both the standard and improved TEM cells, at both selected frequencies of 700 MHz and 3.5 GHz. The spatial region over which homogeneity was evaluated corresponds to the region of the bulk volume or the area of the surface layer at the biological cell level (bottom layer). An overall good homogeneity was observed for the different configurations. At 3.5 GHz, although the electric field is lower (380 vs. 250 V/m), the improved TEM cell achieves a better field homogeneity of ±0.10 dB compared to ±0.50 dB for the standard cell.

### 3.3. SAR Numerical Simulations

**Figure 5:**
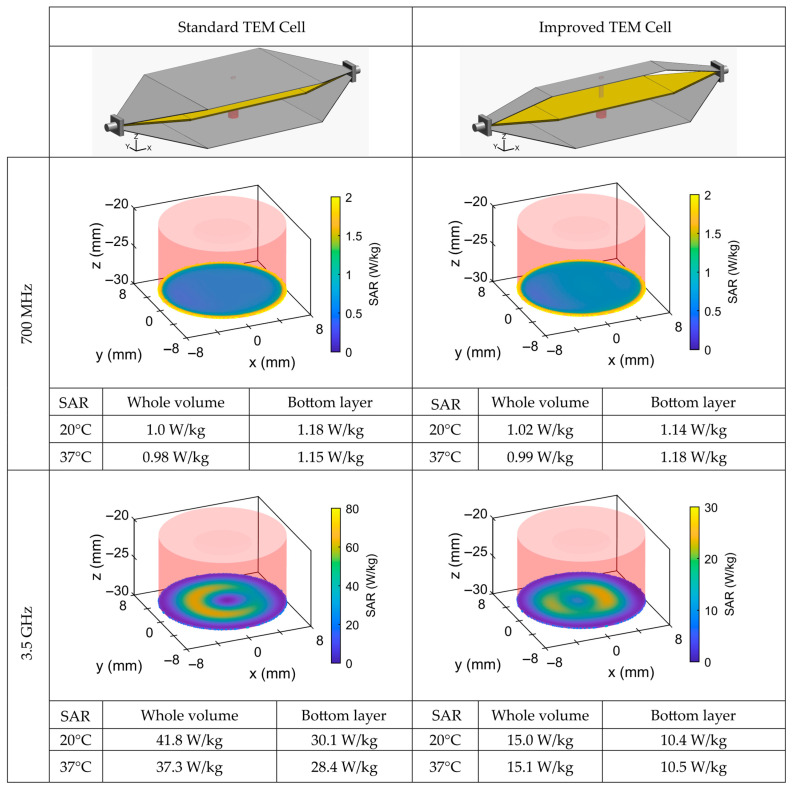
SAR distribution normalized for 1 W of incident power extracted from numerical simulations within the standard and narrow TEM cells at 700 MHz and 3.5 GHz. Plotted distribution corresponds to the bottom layer (surface SAR) of the PTFE chamber, where the biological sample is located. SAR numerical values are computed for both cells (standard and improved) at 20 °C and 37 °C for the whole volume (bulk) and bottom layer (surface).

From numerical simulations, we have extracted the SAR profile for the standard and improved cells at 700 MHz and 3.5 GHz (Figure 5). The plotted profile corresponds to the bottom layer (surface) of the PTFE chamber, where the biological samples are located during BRET experiments. All SAR values are normalized for 1 W of incident power. The incident power corresponds to the net accepted power of the TEM cell at its input after accounting for the reflected power and impedance mismatch.

At 700 MHz, the SAR profile is uniform for both TEM cells with nearly identical bulk and surface SAR values of around 1 W/kg.

Compared to 700 MHz, at 3.5 GHz, the FDTD simulations revealed that the SAR distribution exhibited higher values for both TEM cells. Moreover, significant differences were obtained between bulk- and surface-layer SARs.

Based on these results, the standard TEM cell is better suited for exposures at 700 MHz (achieving SAR values of 1 W/kg), whereas at 3.5 GHz, the improved cell provides more homogeneous exposure, with whole-volume and bottom-layer SAR values of 15 W/kg and 10.5 W/kg, respectively.

For both frequencies and both TEM cells, the SAR was extracted from simulations and experimental measurements at temperatures of 20 °C and 37 °C and showed only minimal dependence on temperature.

### 3.4. SAR Measurements and Thermoregulation

We have conducted experimental SAR assessments based on temperature measurements for both TEM cell configurations and both frequencies (700 MHz and 3.5 GHz). However, based on the numerical SAR distributions, experimental SAR values were ultimately reported only for two cases, i.e., the standard cell at 700 MHz and the improved cell at 3.5 GHz.

The comparison between simulations and measurements was performed using the probe-level SAR, defined within the fluoro-optic measurement volume, estimated at approximately 1 mm^3^. The experimental SAR values, normalized for 1 W of incident power, were 0.7 ± 0.2 W/kg in the standard TEM cell at 700 MHz and 10.0 ± 2.0 W/kg in the improved cell at 3.5 GHz. These values are similar to the equivalent probe SAR extracted from numerical simulations, i.e., 0.7 ± 0.05 W/kg and 12.0 ± 2.0 W/kg, at 700 MHz and at 3.5 GHz, respectively.

**Figure 6:**
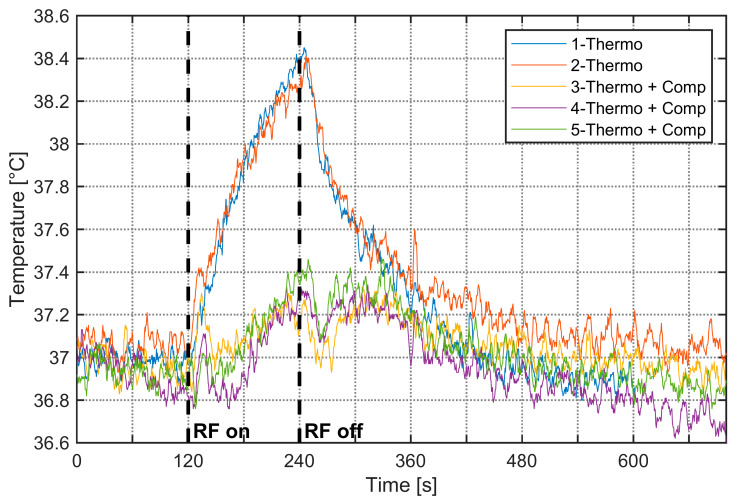
Temperature measurements within the improved cell at 3.5 GHz. Plots #1–2 correspond to 37 °C thermoregulation only, whereas plots #3–5 illustrate temperature variation with additional thermoregulation to compensate for the RF-induced temperature increase; the RF signal is set between 120 s and 240 s with generated power of 20 W.

As RF exposure can heat aqueous samples, strict temperature control is essential when using BRET to study thermo-TRP channels. The setup was, therefore, thermoregulated at 37 °C. Upon activation of the RF signal, a temperature increase was observed specifically at 3.5 GHz. Figure 6 shows the 37 °C thermoregulation measurements performed on the improved cell and the resulting 1.5 °C rise in temperature over 120 s of 3.5 GHz RF exposure for a generated power of 20 W (an equivalent bulk SAR of 300 W/kg). To counteract this increase, the water-circulation thermoregulation system was activated, with effects visible on results #3–5 on Figure 6. In these experiments, we aim to achieve the highest SAR possible while limiting and controlling the temperature elevation. These high SAR values are not typical of biological experiments related to environmental RF exposure studies.

## 4. Discussion and Conclusions

This paper presents the design and characterization of a thermoregulated transverse electromagnetic cell optimized for bioelectromagnetic investigations, particularly for studies on bioluminescence resonance energy transfer at frequencies of 700 MHz and 3.5 GHz. The primary innovation of this work lies in the development of a TEM cell specifically engineered to be compatible with thermoregulation requirements. The cell was designed for BRET measurements at 3.5 GHz, which necessitated several geometric modifications to the conventional TEM architecture. A quantitative comparison of this work with recent designs of wideband TEM cells is provided in Table 4.

We have performed numerical simulations to characterize the electric field and SAR profile in the cell culture medium. Simulations of the electric field profile for the standard and improved TEM cells at 700 MHz and 3.5 GHz show that at 700 MHz, the standard cell has a homogeneous and symmetrical field, while the narrow cell exhibits inhomogeneity and slight standing waves, linked to higher insertion losses (~–1.5 dB). At 3.5 GHz, both cells display inhomogeneities, but the improved cell is relatively uniform in the test volume between the septum and the bottom ground conductor, where biological samples are placed for BRET studies. Numerical simulations show uniform SAR distributions around 1 W/kg for both cells at 700 MHz. At 3.5 GHz, SAR values increase, and their distribution becomes less uniform, with the improved cell providing the most homogeneous exposure with 15 W/kg and 10.5 W/kg for the bulk and surface SARs, respectively.

We have conducted experimental measurements to characterize S-parameters, the SAR profile in the cell culture medium, and temperature for SAR extraction and thermoregulation monitoring.

An enhancement of the TEM cell bandwidth was achieved by modifying the width of the upper outer conductor and optimizing impedance matching at the connector transitions. The experimentally measured results show a significant reduction in return and insertion losses, as well as improved electric field uniformity within the test volume, while maintaining a sufficient usable test volume for biological experiments. At 3.5 GHz, the measured return and insertion losses in the improved cell correspond to approximately −25 dB and −1 dB, respectively. The findings indicate the superior performance of the improved TEM cell, particularly in the 2.5–6 GHz frequency band, due to the suppression of resonance frequencies and higher-order propagation modes.

Experimental SAR measurements were performed on the standard TEM cell at 700 MHz and the improved cell at 3.5 GHz. Probe-level SAR comparisons, using the ~1 mm^3^ fluoro-optic sensing volume, yielded experimental values of 0.7 ± 0.2 W/kg at 700 MHz and 10.0 ± 2.0 W/kg at 3.5 GHz, in good agreement with simulations (0.7 ± 0.05 W/kg and 12.0 ± 2.0 W/kg, respectively).

Because RF exposure can heat aqueous samples, we have thermoregulated the system at 37 °C. At 3.5 GHz, RF activation produced a 1.5 °C rise over 120 s at 20 W (≈300 W/kg whole-volume SAR), which was mitigated using the water-circulation cooling system. Without thermoregulation, to assess the experimental SAR, the temperature elevation is limited by the RF exposure duration to a few degrees, typically 2 degrees. With thermoregulation, the temperature elevation is also reduced to about 1.5 degrees. Under these conditions, the dielectric properties exhibit only minor variations. For an experimental SAR of 300 W/kg, a first-order approximation yields an electric field on the order of 400 V/m, and under such conditions, we can reasonably assume that the system remains within the linear response regime.

We performed an uncertainty analysis following the method for exposure system uncertainty assessment described in [29,30]. The combined uncertainty of the numerical and experimental dosimetry, including the FDTD mesh, dielectric properties and thermal measurements, resulted in a 0.6 dB standard deviation similar to that of [31].

Optimizing the TEM cell at 3.5 GHz inherently introduces a tradeoff, manifested as a slight degradation in the electromagnetic field homogeneity at 700 MHz along the TEM structure, likely due to frequency-dependent modal behavior and impedance-matching constraints. However, the impact of the inhomogeneity is rather limited on the electric field and SAR values at the level of the biological sample.

The results evidence that thermo-regulated TEM cells are suitable for RF exposure and BRET experimental use. Specifically, the improved cell is better adapted for exposures at 3.5 GHz, whereas the standard TEM cell demonstrates good performance at 700 MHz in terms of return and insertion losses, as well as electric field and SAR homogeneity.

The SAR values are not typical of biological experiments related to environmental RF exposure studies regulated by international guidelines. The system allows us to explore markedly elevated exposure conditions, with strict control of temperature rise to prevent confounding thermal effects.

This study provides a solid foundation for future research on the effects of 5G signals on biological cells, especially in the context of BRET investigations. The authors also emphasize the importance of thermoregulation in biological experiments and propose an integrated cooling system to maintain a constant temperature in the cell culture medium.

In conclusion, this thermoregulated, bandwidth-enhanced TEM cell represents a significant advancement for bioelectromagnetic studies, offering an effective solution for 5G 3.5 GHz signal exposure while ensuring optimal experimental conditions for biological samples. The results pave the way for further research into the interactions between electromagnetic fields and biological systems.

## Figures and Tables

**Table 1:** Detailed list of the components of the BRET investigation setup.

Item	Brand	Model	Additional Information
RF Generator	Rohde & Schwarz, Munich, Germany	SMBV100A	5G (9 kHz–6 GHz)
Power Supply	ITech, New Taipei City, Taiwan	IT6722A	0–80 V/20 A
Amplifier	Mini-circuits, Brooklyn, NY, USA	ZHL0G60G7100+	600–700 MHz
Circulator	Pasternack, Irvine, CA, USA	PE8419	698–960 MHz
Dir. Coupler	Mini-circuits	ZGBDC30-372HP+	380–3700 MHz; 30 dB
Power Meter	Agilent, Santa Clara, CA, USA	E4417A with E9325A	EPM-P Series
Attenuator	Sodhy, Verrieres-le-Buisson, France	AT 200-30dB-N	30 dB
Data Acq.	Agilent	34901A & 34970A	20 channels
Temperature Acq.	Luxtron-Lumasense, San Jose, CA, USA	Model 812	2 Fluoro-optic probes
Spectrometer	Teledyne-Princeton Instruments, Lisses, France	IsoPlane SCT-320	BLAZE:400B CCD

**Table 2:** PBS electrical properties considered in numerical simulations.

	700 MHz		3.5 GHz	
Temperature	20 °C	37 °C	20 °C	37 °C
Relative permittivity, ε_r_	75	73	74.2	71.8
Conductivity, σ (S/m)	1.73	1.57	2.3	3.3

**Table 3:** Quantitative metrics characterizing the electric field distribution (mean value ± dB).

	700 MHz		3.5 GHz	
TEM Cell	Bulk region	Surface area	Bulk region	Surface area
Standard	366.1 V/m ± 0.02 dB	364.9 V/m ± 0.02 dB	383.7 V/m ± 0.50 dB	382.3 V/m ± 0.50 dB
Improved	361.9 V/m ± 0.02 dB	360.7 V/m ± 0.01 dB	254.1 V/m ± 0.10 dB	255.4 V/m ± 0.10 dB

**Table 4:** Quantitative comparison of the present work with other recent TEM cell designs from the literature.

Ref.	Measured Bandwidth (GHz) ${\left|S_{11}\right|}_{dB}<-10\;dB$	Test Volume (${mm}^{3}$) (Septum Width × Septum Length × Septum-Ground Spacing)
[26]	3.0	30 × 100 × 12
[27]	1.8	114 × 150 × 45
[4]	1.8	131 × 400 × 110
[28]	9.6	17 × 31 × 12
This work	6.0	80 × 200 × 30

## Data Availability

The original contributions presented in this study are included in the article. Further inquiries can be directed to the corresponding author.

## Abbreviations

The following abbreviations are used in this manuscript:

TEM cell	Transverse electromagnetic cell
BRET	Bioluminescence resonance energy transfer
SAR	Specific absorption rate
FDTD	Finite-difference time domain
PTFE	Polytetrafluoroethylene
